# Sepsis Among Hospitalized Patients: Events in the 30 Days Preceding the Diagnosis

**DOI:** 10.7759/cureus.84983

**Published:** 2025-05-28

**Authors:** Catherine A Marco, Tori Beth L Snoad, Hali Kim, Quincy Erturk, Kayla Keenan, Grace Wang, Eric Hu

**Affiliations:** 1 Emergency Medicine, Penn State Health Milton S. Hershey Medical Center, Hershey, USA; 2 Emergency Medicine, Penn State College of Medicine, Hershey, USA

**Keywords:** emergency sepsis, emergency service, healthcare encounters, infectious diseases epidemiology, sepsis, septic shock

## Abstract

Introduction

Sepsis is defined as life-threatening organ dysfunction due to a dysregulated host response to infection, and is one of the leading causes of morbidity and mortality globally. This study was undertaken to investigate the role of healthcare encounters and diagnostic test results in the 30 days preceding a sepsis diagnosis.

Methods

In this single-center retrospective chart review, eligible subjects were 18 years of age and older who presented to the ED and were diagnosed with either sepsis or septic shock in the ED or inpatient setting between January 2020 and September 2023. Healthcare encounters such as laboratory and diagnostic studies obtained within 30 days prior to the diagnosis of sepsis or septic shock were included in the analysis.

Results

A total of 363 ED patients with a diagnosis of sepsis were included in the study. The mean age of the patients was 64 years (range 18-99), with 47% being women. At ED presentation, 202 (66%) had an abnormal chest radiograph, and 224 (75%) had an abnormal EKG. Final disposition included discharge home from inpatient setting (n=171; 48%), deceased (n=63; 17.55%), and transfer to an extended care facility (ECF) or rehabilitation center (n=125; 34.82%). A substantial number had a healthcare encounter within the previous 30 days prior to the diagnosis of sepsis (n=131; 36%; range: 1-28). Types of healthcare encounters included ED visit (n=113; 31%), inpatient hospitalization (n=85; 23%), outpatient visit (n=77; 21%), ECF (n=5; 1.2%), and home health visit (n=2; 0.5%).

Patients with more encounters within 30 days had higher mortality (deceased patients had a mean of 4.6 visits (95%CI: 3.0, 6.1), and patients discharged home had a mean of 3.0 visits (95%CI (2.3, 3.6) (p = 0.03). Older patients were more likely to be transferred to an ECF/rehabilitation center than discharged home (mean age of patients transferred to ECF: 70.9, 95%CI 68.4, 73.4; mean age of patients discharged home: 58.6, 95%CI 55.7, 61.5; p<0.0001).

Conclusions

Patients with a diagnosis of sepsis frequently had one or more healthcare encounters and diagnostic tests within 30 days prior to the diagnosis. Abnormal diagnostic tests, including creatinine, bilirubin, and alkaline phosphatase, were associated with higher mortality. Patients with more healthcare encounters and older patients had higher mortality.

## Introduction

Sepsis is defined as life-threatening organ dysfunction due to a dysregulated host response to infection [1,2]. Sepsis is one of the leading causes of morbidity and mortality globally, accounting for 48.9 million patients with an associated sepsis-related death rate of 20% [3-6]. Of importance, around 850,000 sepsis cases present to emergency departments (EDs) in the United States every year, rendering EDs “a main point of entry for patients with sepsis” into hospitals [7,8]. In the ED, many patients admitted with a diagnosis of sepsis first present as medical emergencies [9]. Several studies have shown that early identification and treatment reduce sepsis mortality [10,11]. Multiple initiatives have been developed in attempts to improve diagnostic accuracy and timely treatment of sepsis, with the goals of improved morbidity and mortality [12-16].

Useful predictive models have shown that independent risk factors for sepsis in patients with urinary tract infections (UTI) include, although not limited to, heart failure, diabetes, liver disease, fluid electrolyte disorders, Acute Physiology Score III (APSIII score), neutrophils, lymphocytes, red blood cell distribution width, urinary protein, urinary blood, and microorganisms [17]. Additionally, poor outcomes are associated with thrombocytopenia, high neutrophil counts, and elevated levels of bilirubin, urea, presepsin, and procalcitonin in sepsis patients [18]. Despite efforts to improve the diagnosis of sepsis, the role of preceding diagnostic studies has yet to be explored and understood.

Sepsis is a devastating diagnosis for patients, their families, and hospital systems in their own respects. This study was undertaken to investigate the role of healthcare encounters and diagnostic test results in the 30 days preceding a sepsis diagnosis.

## Materials and methods

This was a single-center retrospective chart review conducted at Penn State Health Milton S. Hershey Medical Center, Hershey, Pennsylvania, United States. The study was approved by the Penn State University Institutional Review Board. The study did not require formal IRB review because it met the criteria for exempt research according to the policies of the institution and the provisions of applicable federal regulations.

All patients aged 18 years and older who presented to the ED and were diagnosed with either sepsis or septic shock in the ED or inpatient setting between January 2020 and September 2023 were included in the study. Exclusion criteria included patients under the age of 18. 

Chart reviews were performed by trained researchers. Methodologic standards included abstractor training, case selection criteria, abstraction forms, and performance monitoring (10% of charts were reviewed by a second researcher). Chart abstractors were not blinded to the study hypothesis. 

Data were analyzed using SAS software version 9.4 (Released 2023; SAS Institute Inc., Cary, North Carolina, United States). Statistical p values of <0.05 were considered significant. Comparisons between groups were performed with ANOVA and Chi-Square tests. 

## Results

A total of 363 ED patients with a diagnosis of sepsis were included in the study. The mean age of patients was 64 years (range 18-99), with 47% being women. Final disposition included discharge home (n=171; 48%), deceased (n=63; 17.55%), and transfer to an extended care facility (ECF) or rehabilitation center (n=125; 34.82%). There were four subjects with missing data for final disposition.

A substantial number of patients had a healthcare encounter within the 30 days prior to the diagnosis of sepsis (n=131; 36%; range 1-28) (Table 1). Types of healthcare encounters included ED visit (n=113; 31%), inpatient hospitalization (n=85; 23%), outpatient visit (n=77; 21%), ECF (n=5; 1.2%), and home health visit (n=2; 0.5%). (Table 2). A minority of patients had a healthcare encounter within one day of the diagnosis of sepsis (n=24; 21%). Patients with more healthcare encounters within 30 days had higher mortality (deceased patients had a mean of 4.6 visits (95%CI 3.0, 6.1) and patients discharged home had a mean of 3.0 visits (95%CI 2.3, 3.6) (p=0.03) (Table 3).

**Table 1:** Distribution of patients according to healthcare encounters within the 30 days prior* to the diagnosis of sepsis (total number of patients=363) *excluding ED visit on the day of sepsis diagnosis

Number of healthcare encounters	Number of Patients (Percentage)
0	232 (63.91%)
1	21 (5.79%)
2	19 (5.23%)
3	11 (3.03%)
4	10 (2.75%)
5	5 (1.38%)
6	8 (2.20%)
≥7	57 (15.70%)

**Table 2:** Patients who had healthcare encounters within the 30 days prior to the diagnosis of sepsis distributed according to type of encounter (N=131)* * The total number of healthcare encounters is 282 because some of the 131 patients had more than one encounter. ECF: extended care facility

Type of encounter 30 days prior to sepsis diagnosis	Number of patients	Patients who received antibiotics in the 30 days prior to the diagnosis of sepsis, n (%)
Emergency Department	113	59 (45.03%)
Inpatient	85	40 (30.53%)
Outpatient	77	12 (9.16%)
ECF	6	3 (2.29%)
Home Health	2	0 (0%)

**Table 3:** Disposition of 359 patients* with sepsis and association with number of previous healthcare encounters *Of 363 total participants, four had missing data for disposition. ECF: extended care facility

Disposition (Outcome)	Number of patients (percentage)	Number of previous healthcare encounters within 30 days, mean (95% CI)	t-test statistic, p-value
Discharge home	171 (47.63%)	3.0 (2.3-3.6)	2.20, 0.029
Transfer to ECF	125 (34.81%)	3.6 (2.7-4.4)	1.02, 0.31
Deceased	63 (17.55%)	4.6 (3.0-6.1)	1.32, 0.19

Age was significantly associated with outcomes. The mean age of deceased patients was 67.2 (95%CI 63.2, 71.2) and the mean age of patients discharged home was 58.6 (95%CI 55.7, 61.5) (p=0.0007) (Table 4). Older patients were more likely to be transferred to an ECF/rehabilitation center than discharged home (patients transferred to ECF had a mean age of 70.9 years (95%CI 68.4, 73.4); patients discharged home had a mean age of 58.6 (95%CI 55.7, 61.5) (p<0.0001). There was no statistical significance associated with disposition type with gender, ED triage heart rate, systolic blood pressure, or respiratory rate (Tables 5-8).

**Table 4:** Disposition of 359 patients* with sepsis and association with age *Of 363 total participants, four had missing data for disposition. ECF: extended care facility

Disposition (Outcome)	Number of patients (percentage)	Age, mean (95% CI)	t-test statistic, p-value
Discharge home	171 (47.63%)	58.6 (55.7-61.5)	3.41, 0.0007
Transfer to ECF	125 (34.81%)	70.9 (68.4-73.4)	6.13, <0.0001
Deceased	63 (17.55%)	67.2 (63.2-71.2)	-1.41, 0.158

**Table 5:** Disposition of 359 patients* with sepsis and association with sex *Of 363 total participants, four had missing data for disposition. ECF: extended care facility

Disposition (Outcome)	Number of patients (percentage)	Sex (male), n (%)	Chi-Square statistic, p-value
Discharge home	171 (47.63%)	86 (50.3%)	0.75, 0.69
Transfer to ECF	125 (34.81%)	69 (55.2%)	0.75, 0.69
Deceased	63 (17.55%)	34 (54%)	0.75, 0.69

**Table 6:** Disposition of 359 patients* with sepsis and association with triage heart rate *Of 363 total participants, four had missing data for disposition. ECF: extended care facility

Disposition (Outcome)	Number of patients (percentage)	Triage heart rate, mean (95% CI)	t-test statistic, p-value
Discharge home	171 (47.63%)	99.3 (95.8-102.8)	-0.97, 0.33
Transfer to ECF	125 (34.81%)	96.8 (93.1-100.4)	0.70, 0.48
Deceased	63 (17.55%)	99.2 (93.5-104.9)	-0.03, 0.98

**Table 7:** Disposition of 359 patients* with sepsis and association with triage systolic blood pressure *Of 363 total participants, four had missing data for disposition. ECF: extended care facility

Disposition (Outcome)	Number of patients (percentage)	Triage blood pressure, mean (95% CI)	t-test statistic, p-value
Discharge home	171 (47.63%)	120.1 (116.1-124.1)	-0.30, 0.76
Transfer to ECF	125 (34.81%)	118.5 (113-124)	-0.48, 0.63
Deceased	63 (17.55%)	118.9 (112.1 – 125.6)	0.08, 0.94

**Table 8:** Disposition of 359 patients* with sepsis and association with triage respiratory rate *Of 363 total participants, four had missing data for disposition. ECF: extended care facility

Disposition (Outcome)	Number of patients (percentage)	Triage respiratory rate, mean (95% CI)	t-test statistic, p-value
Discharge home	171 (47.63%)	21.4 (20-22.9)	0.13, 0.90
Transfer to ECF	125 (34.81%)	22 (19.5-24.5)	0.43, 0.67
Deceased	63 (17.55%)	21.7 (20-23.3)	-0.20, 0.84

Abnormal creatinine within the previous 30 days was associated with higher mortality (Table 9). ED Laboratory markers were associated with outcomes. Patients with higher blood urea nitrogen (BUN) levels were more likely to be deceased than discharged home (deceased patients’ mean BUN 39.6, 95%CI 32.4, 46.8; patients discharged home had mean BUN 31.5, 95%CI 27.5, 35) (p=0.0425). Those with higher bilirubin levels were more likely to be deceased compared to being transferred to an ECF/rehabilitation center (deceased patients’ mean bilirubin 3.0, 95%CI 1.3, 4.8; patients transferred to ECF had mean bilirubin 1.0, 95%CI 0.7, 1.3) (p=0.0002), and being discharged home (deceased patients’ mean bilirubin 3.0, 95%CI 1.3,4.8; patients discharged home had mean bilirubin 1.2, 95%CI 0.8, 1.5) (p=0.0003). Those with higher ED alkaline phosphatase were more likely to be deceased compared to being transferred to an ECF/rehabilitation center (deceased patients’ mean alkaline phosphatase 208.9, 95%CI 132.5, 285.3; patients transferred to ECF had mean alkaline phosphatase 136.5, 95%CI 114.0, 159.0) (p=0.0216). The values for abnormal urinalysis, chest X-ray, and ECG in previous healthcare encounters among the patients diagnosed with sepsis are given in Table 10.

**Table 9:** Laboratory markers in previous encounters among the patients with healthcare encounters within 30 days prior to the diagnosis of sepsis (N=131) ECF: extended care facility

Laboratory markers	Deceased, mean (95% CI)	Discharged to ECF, mean (95% CI)	Discharged home, mean (95% CI)	ANOVA, p-value
WBC	15.5 (11.1-19.8)	15.6 (13.1-18.1)	18.6 (12.9-24.4)	F (2 ,226) = 0.51, p=0.60
Sodium	138 (136-139)	138 (137-139)	138 (137-139)	F (2 ,158) = 0.09, p=0.92
Potassium	4.1 (3.9-4.3	4.2 (4-4.3)	4.2 (4-4.2)	F (2 ,158) = 0.22, p=0.80
Creatinine	2.6 (0.6-4.6)	1.5 (1-1.9)	1.3 (1.1-1.5)	F (2 ,155) = 3.11, p=0.05
Glucose	126 (106-145)	157 (133-180)	134 (120-149)	F (2 ,158) = 2.42, p=0.09
Bilirubin	1.8 (0.03-3.9)	1.4 (0.5-2.2)	1.3 (0.7-2)	F (2 ,114) = 0.21, p=0.81
Alkaline phosphatase	279 (76-481)	176 (67-285)	181 (127-236)	F (2 ,113) = 0.98, p=0.38

**Table 10:** Abnormal urinalysis, chest X-ray, and ECG in previous encounters among the patients with healthcare encounters within 30 days prior to the diagnosis of sepsis

Parameters	Deceased, percentage (95% CI)	Discharged to ECF, percentage (95% CI)	Discharged home, percentage (95% CI)	Chi-square, p-value
Abnormal urinalysis	7% (0.4-14)	10% (4-16)	12% (7-17)	χ2 (2, N = 321) = 1.15, p=0.56
Abnormal chest radiograph	11% (2.7-19.9)	8% (3.1-13.7)	12% (6.5-16.7)	χ2 (2, N = 315) = 0.74, p=0.69
Abnormal ECG	8% (0.4-15.3)	13% (6.3-18.7)	11* (6.1-16.2)	χ2 (2, N = 315) = 0.77, p=0.68

## Discussion

Sepsis is a life-threatening condition. Early recognition and diagnosis of sepsis is crucial to early treatment, which has been associated with better outcomes. Gatewood et al. showed that early screening interventions led to early treatment for patients with sepsis, and mortality rates fell, though not significantly [19]. Similarly, Torsvik et al. found that early sepsis recognition, which included severity, vital signs, and treatment, led to increased 30-day survival, lower risk of developing severe organ failure, and shorter length of stay [20]. 

In this study, it was found that patients who had more previous healthcare encounters within 30 days prior to sepsis diagnosis were more likely to be deceased than discharged home. This result is in accordance with the systematic review by Flannery et al., which stated that 32.7% of patients on average have an encounter with the healthcare system in the week prior to a sepsis hospitalization [21]. Similarly, Liu et al. found that over 45% of sepsis patients had healthcare encounters in the week prior to hospitalization, with a high rate of diagnosis for acute infection and antibiotic use in the outpatient setting [22]. This may be due to an association of previously undiagnosed illness with the subsequent development of sepsis. This suggests a potential opportunity for early recognition, diagnosis, and treatment.

There is extensive research on biomarkers as potential predictors or having associations with sepsis diagnosis, two of the most common being C-reactive protein and procalcitonin [23]. The current study found that high bilirubin was associated with a higher risk of mortality compared to either being discharged home or transferred to an ECF. This finding corroborates that of previous studies, including a study by Patel et al., who found elevated bilirubin levels to be associated with increased risk of mortality in severe sepsis and septic shock [24], and another by Peng et al., who found similar results with potential for setting cutoff values for early treatment [25].

Rapid diagnosis of patients with sepsis is essential to timely management. Antimicrobial agents and intravenous fluids should be administered expeditiously to prevent delays associated with increased mortality. Vasopressors should be considered for patients with hypotension [26]. Many EDs have initiated sepsis alert systems to identify patients at risk for sepsis and expedite treatment [27].

Limitations

This study included data from a single institution and may not be generalizable to other settings. This study analyzed chart review data and is dependent on the accuracy of medical records. Because our data included only data from our healthcare system, the actual number of healthcare encounters and diagnostic tests may have been higher than reported.

## Conclusions

This study found that patients with a diagnosis of sepsis frequently had one or more healthcare encounters and diagnostic tests within 30 days prior to the diagnosis. Patients with more healthcare encounters and older patients had higher mortality. Abnormal diagnostic tests, including creatinine, bilirubin, and alkaline phosphatase, were associated with higher mortality. These results should aid in the identification of patients at high risk for morbidity and mortality and aid in expeditious treatment to prevent complications of sepsis.
